# New Insights in Cushing Disease Treatment With Focus on a Derivative of Vitamin A

**DOI:** 10.3389/fendo.2018.00262

**Published:** 2018-05-24

**Authors:** Mariana Fuertes, Julieta Tkatch, Josefina Rosmino, Leandro Nieto, Mirtha Adriana Guitelman, Eduardo Arzt

**Affiliations:** ^1^Instituto de Investigación en Biomedicina de Buenos Aires (IBioBA) – CONICET – Partner Institute of the Max Planck Society, Buenos Aires, Argentina; ^2^División Endocrinología, Hospital General de Agudos “Carlos G. Durand”, Buenos Aires, Argentina; ^3^Departamento de Fisiología y Biología Molecular y Celular, Facultad de Ciencias Exactas y Naturales, Universidad de Buenos Aires, Buenos Aires, Argentina

**Keywords:** Cushing disease, pharmacological treatment, adrenocorticotropic hormone, retinoic acid, chicken ovoalbumin upstream promoter transcription factor

## Abstract

Cushing’s disease (CD) is an endocrine disorder originated by a corticotroph tumor. It is linked with high mortality and morbidity due to chronic hypercortisolism. Treatment goals are to control cortisol excess and achieve long-term remission, therefore, reducing both complications and patient’s mortality. First-line of treatment for CD is pituitary’s surgery. However, 30% of patients who undergo surgery experience recurrence in long-term follow-up. Persistent or recurrent CD demands second-line treatments, such as pituitary radiotherapy, adrenal surgery, and/or pharmacological therapy. The latter plays a key role in cortisol excess control. Its targets are inhibition of adrenocorticotropic hormone (ACTH) production, inhibition of adrenal steroidogenesis, or antagonism of cortisol action at its peripheral receptor. Retinoic acid (RA) is a metabolic product of vitamin A (retinol) and has been studied for its antiproliferative effects on corticotroph tumor cells. It has been shown that this drug regulates the expression of pro-opiomelanocortin (POMC), ACTH secretion, and tumor growth in corticotroph tumor mouse cell lines and in the nude mice experimental model, *via* inhibition of POMC transcription. It has been shown to result in tumor reduction, normalization of cortisol levels and clinical improvement in dogs treated with RA for 6 months. The orphan nuclear receptor COUP-TFI is expressed in normal corticotroph cells, but not in corticotroph tumoral cells, and inhibits RA pathways. A first clinical human study demonstrated clinical and biochemical effectiveness in 5/7 patients treated with RA for a period of up to 12 months. In a recent second clinical trial, 25% of 16 patients achieved eucortisolemia, and all achieved a cortisol reduction after 6- to 12-month treatment. The goal of this review is to discuss in the context of the available and future pharmacological treatments of CD, RA mechanisms of action on corticotroph tumor cells, and future perspectives, focusing on potential clinical implementation.

## Introduction

Cushing’s disease (CD) is a severe endocrine disorder associated with increased morbidity and mortality, caused by an adrenocorticotropic hormone (ACTH) secreting pituitary adenoma (1). It is the most common form of endogenous Cushing’s syndrome (CS) (2), and it is the consequence of excessive secretion of cortisol from the adrenal glands. ACTH pituitary adenomas are microadenoma in more than 90% of cases (3).

The clinical phenotype involves features related to metabolic syndrome (diabetes mellitus, central obesity, hypertension, and dyslipidemia), muscle weakness, easy bruising, hirsutism, and cognitive disorders. The main cause of death is represented by cardiovascular disease (4–6).

First-line of treatment for CD is transsphenoidal surgery, aimed to remove the pituitary adenoma. However, success rates are variable (reported as 65–90%) depending on the surgeon’s expertise (5, 7). Pituitary radiotherapy and bilateral adrenalectomy are usually selected as second-line therapies when hypercortisolism persists; however, they are associated with long-term complications (6, 8).

Pharmacological treatment is recommended to control cortisol excess when surgery is contraindicated or non-curative, or while waiting for radiotherapy to come into effect (9, 10).

In this review, we will summarize the current state of art of pharmacological treatment of CD. We will focus on retinoic acid (RA), its molecular mechanism of action in different models of CD, its use, and future perspectives.

### Pharmacological Therapy of CD

There are three pathophysiological mechanisms that are targeted by different treatments: central inhibition of ACTH secretion, adrenal-directed inhibition of steroidogenesis, and glucocorticoid receptor (GR) blockade (11) (Table 1).

**Table 1:** Summary of pharmacological therapy and clinical trials of CD.

	Human clinical trial	Effectiveness	Side effects	Condition

Central inhibition of adrenocorticotropic hormone secretion drugs				
Pasireotide	Multicenter phase III trial (12)	15 and 26% of 162 patients with a dose of 600 and 900 μg/twice daily subcutaneous	Hyperglycemia, diarrhea, nausea, cholelithiasis, headache, abdominal pain	Approved

	Phase III trial (3)	All patients (8) with significant tumor shrinkage		

	Prospective clinical trial (13)	Biochemical and clinical improvement after 5 years		

	Prospective phase III trial (14)	40% of 150 patients normalized mean urinary free cortisol (UFC) in 7 months with long-acting release pasireotide monthly intramuscular		

Cabergoline	Retrospective, non-randomized analysis (15)	Long term: 30% of 30 patients (mean dose 2.1 mg/week) and short term: 30–40% of 37 patients (mean dose 3.5 mg/week) normalized UFC	Mild (transient dizziness and nausea)	Off-label

	Prospective, single center study (16)	No significant UFC reduction in 20 patients (mean dose 5 mg/week)		

	Retrospective, multicenter trial (17)	40% of 53 patients normalized UFC		

Rosiglitazone	*In vivo* study (18)	30–43% of patients normalized UFC (4–16 mg/day)	Hepatotoxicity and cardiotoxicity	Out of medical market

**Adrenal-directed inhibition of steroidogenesis drugs**				

Ketoconazole	French retrospective multicenter study (19)	49.3% of 200 patients achieved normal UFC and 25.6% had at least a 50% decrease	Increment in liver enzymes. Potential fatal liver injuries and possible drug interactions	Off-label, limited use

Metyrapone	Retrospective study (20)	43–76% of 164 patients controlled cortisol levels	Gastrointestinal adverse effects and hypoadrenalism	Off-label, approved in the European Union

Mitotane	Retrospective analysis (21)	70% of 76 patients normalized UFC	Neurological side effects, anorexia, hypercholesterolemia, gastrointestinal symptoms, and abnormal liver function	

**Glucocorticoid receptor blockade drugs**				

Mifepristone	Multicenter, open-label trial (22)	60% of 50 patients improved glycemic parameters; 38% reduced diastolic blood pressure	Arthralgia, nausea, headache, peripheral edema, decreased blood potassium, fatigue, and endometrial thickening	Approved by Food and Drug Administration to patients with hyperglycemia or type 2 diabetes mellitus

**New molecular target drugs**				

Osilodrostat	Proof-of-concept study (23)	92% of 12 patients normalized UFC at 10 weeks	Fatigue, nausea, and headache	

	Phase II trial (23)	84 and 79% of patients normalized UFC at 10 or 22 weeks, respectively		

	2 Phase III studies	Ongoing		

Levoketoconazole	Phase III single-arm, open-label trial	Ongoing		

	Phase III clinical trial	Ongoing		

R-roscovitine	Phase II clinical trial	Ongoing		

**Retinoic acid**				

	Prospective, multicenter study (24)	43% of 7 patients normalized UFC (10–80 mg/day for 6–12 months)	Conjunctival irritation, headache, arthralgias, nausea	Approved for acne

	Prospective trial (25)	25% of 16 patients normalized UFC and salivary cortisol (20–80 mg/day for 6–12 months)	Conjunctiva irritation, cheilitis, mucositis, nausea, headache, arthralgias	

	Prospective, single-arm, open-label clinical trial	Ongoing		

We will also discuss new molecular targets and other medical treatment perspectives. The availability of drugs with different mechanisms of action expand the therapeutic spectrum and allow drug combinations, as will be pointed, thus permitting cortisol levels normalization with the lowest rate of adverse effects.

#### Central Inhibition of ACTH Secretion

Corticotroph adenomas frequently express both dopamine receptors (DRs) and somatostatin transmembrane receptor (SSTR).

##### Somatostatin Receptor Ligands (SRLs)

Pasireotide (SOM230) is a novel somatostatin analog that binds to multiple SSTR with high affinity and has a 40-fold higher affinity for SSTR_5_ than octreotide. SSTR_5_ is the predominant SSTR expressed in corticotroph tumors (7, 26). Octreotide and lanreotide have high affinity for SSTR_2_ and moderate affinity for SSTR_5_. The expression of SSTR_2_ is reduced by hypercortisolism and SRLs acting on SSTR_2_ are not effective in CD patients (27, 28). SSTR_5_ expression may be less susceptible to the suppressive effect of cortisol (29–31).

Pasireotide is currently the only approved treatment for CD directed toward the pituitary gland. It was approved in 2012 in US and Europe for the treatment of adult CD patients who did not achieve remission after surgery or when it has been contraindicated (7, 28, 32). This drug has been shown to suppress both ACTH secretion and cell proliferation (11). A multicenter phase III study reported an effectiveness of this somatostatin analog in the subcutaneous formulation in 15 and 26% of a total of 162 patients with a dose of 600 and 900 µg/twice daily (12).

Hyperglycemia is the most common adverse effect occurring in 73% of the patients (12). Pasireotide-induced hyperglycemia is mediated by a reduction of insulin and incretin hormones secretion. Blood glucose levels must be monitored during pasireotide administration, mainly in patients with previous diabetes mellitus or impaired fasting blood glucose (12, 26). Diarrhea, nausea, cholelithiasis, headache, and abdominal pain are also common adverse events (31, 33). A subsequent phase III trial, on eight patients treated in a single center with pasireotide for 6 months, also indicated significant tumor shrinkage (3).

In a prospective, multicenter trial, pasireotide was given alone or in combination with cabergoline (CAB) with or without ketoconazole (KCN) (30). Additional medical therapy in combination with pasireotide may be of benefit for those patients in whom normalization of cortisol levels are not achieved (12, 30).

In a recent prospective clinical trial, a subset of patients with CD treated with pasireotide showed biochemical and clinical improvement after 5 years (13). This study suggests that pasireotide is an effective drug for the long-term treatment of patients with CD, especially when surgery fails or is contraindicated (13).

In the first prospective phase III trial of monthly intramuscular long-acting release pasireotide (LAR), pasireotide normalized mean urinary free cortisol (UFC) in about 40% of 150 patients with CD at month 7 and showed a similar safety profile than twice-daily subcutaneous pasireotide (14). LAR formulation and monthly administration is an efficient option in patients with CD persistent or recurrent, although has not yet been approved (14).

##### DR Agonists

Dopamine receptors are widely expressed in normal neuroendocrine tissues and pituitary adenomas, around 80% corticotroph tumors express dopamine receptor 2 (D2) (34). The DR family consists of five receptor subtypes that include D1-like (D1 and 4) and D2-like (D2, 3, and 4) receptors (6). In corticotroph adenomas, the suppressive action of the dopamine agonist CAB on ACTH secretion in CD patients has a positive correlation with the expression of D2 (34).

In a retrospective, non-randomized analysis of 30 CD patients treated for long term with CAB (mean dose 2.1 mg/week), nine patients (30%) showed a complete response after a mean of 37 months. Two patients did not respond after 2 and 5 years, respectively, but one patient transiently renormalized UFC after increase of CAB dosage. Four initial partial responders did not sustain a long-term response. Adverse effects were mild and included transient dizziness and nausea (15).

A response rate of approximately 30–40% at a high mean dose of 3.5 mg/week (0.5–7 mg/week) has been reported in CD patients (11, 15, 16, 35). Recent studies combining CAB and KCN showed that up to 79% of patients achieved normal levels of UFC (35). Unfortunately, since neither drug has been tested in controlled clinical trials, both are used as off-label therapy for the treatment of CD.

In a prospective single center study (16), 20 patients with CD were treated with CAB (median dose 5 mg/week) during a 6-week period. Prolactin levels were suppressed in all patients (demonstrating treatment compliance). Even though it had been previously suggested that higher doses of CAB improve hypercortisolism, no significant UFC reductions were observed in this trial, thus demonstrating a limited value of the use of CAB for the treatment of CD.

In a retrospective multicenter trial, 53 patients with CD received CAB as mono therapy and 9 patients received CAB complementary to other therapies. UFC normalization was observed in 21 of 53 patients who received CAB as monotherapy during a 12-month period (responding patients); 4 patients were considered partial responders and 28 patients did not modify or raise UFC levels. Among the responding patients, five developed corticoid insufficiency. CAB was withdrawn after 12 months in 28% of the responding patients, due to intolerance or escape. Hypercortisolism control was achieved in 23% of the overall patients, for 32.5 months. No significant differences were observed between baseline UFC levels, but the responding group received lower doses of CAB in comparison to non-responding patients (1.5 vs. 3.5 mg/week). Adequate response and tolerance to CAB was achieved in 20–25% of CD patients and basal UFC levels did not adequately predict the treatment response (17).

##### Peroxisome Proliferator-Activated Receptor (PPAR) Gamma Ligands

Peroxisome proliferator-activated receptor gamma ligands have shown antiproliferative and apoptotic activity in corticotropina murine models. *In vivo* studies using rosiglitazone from 4 to 16 mg/day in CD patients demonstrated UFC normalization between 30 and 43% of treated patients. Due to its hepatotoxicity and cardiotoxicity, this drug was taken off the medical market (18, 36, 37).

#### Adrenal-Directed Inhibition of Steroidogenesis

Adrenal steroidogenesis inhibitors are used as a cornerstone of medical treatment of CD (38). KCN, mitotane, etomidate, metyrapone, aminoglutethimide, and trilostane are drugs that inhibit adrenocortical steroidogenesis. Adrenal steroidogenesis inhibitors are generally used to control hypercortisolism in the short term. These drugs have the disadvantage of not acting directly on the pituitary tumor.

##### Ketoconazole

Ketoconazole, an antifungal substance, is a synthetic derivative of imidazole that diminishes adrenal steroidogenesis (400–1,200 mg). It is one of the most commonly used off-label drug in the treatment of CS (39). KCN blocks key cytochrome P450 (CYP) enzymes that participate in adrenal cortex steroidogenesis. In CS patients, it significantly reduces UFC with a 30–80% efficacy (28, 39).

In a French retrospective multicenter study, in 200 patients with active CD, KCN was administrated as monotherapy. In this study, 49.3% of patients achieved normal UFC, 25.6% had at least a 50% decrease, and in 25.4% UFC levels were unchanged. About half of the patients who received KCN before surgery (40 patients, median final dose of 600 mg/day) exhibited an improvement in hypokalemia, hypertension, and diabetes. Only 13.5 and 2.5% of patients shown mild and major increase in liver enzymes, respectively, that disappeared after drug withdrawal (19).

In 2013, the Food and Drug Administration (FDA) limited its use because of potential fatal liver injuries and possible drug interactions (40). However, the European Agency EMA had recommended a permission of a marketing authorization for KCN (HRA Pharma) in the treatment of CS.

##### Metyrapone

Metyrapone shows potent and selective inhibition of CYP11B1. Reductions in aldosterone and UFC were observed following metyrapone treatment. Gastrointestinal adverse effects and hypoadrenalism have been described. Metyrapone is employed clinically as an off label drug for CS treatment (20, 41).

In a retrospective study with 164 CS patients treated with metyrapone monotherapy, 43–76% of them managed to control cortisol levels. No escape was reported (20). In the European Union, it is approved for the treatment of CS (38).

##### Mitotane (OPDD)

Mitotane, a synthetic derivative of the pesticide dichlorodiphenyltrichloroethane (Lysodren^®^), is mainly used for its adrenolytic action in adrenocortical cancer. It has also a slow, but potent, inhibitory action on steroid biosynthesis when used at lower doses. Adrenal insufficiency (AI) is frequently observed, and replacement therapy with hydrocortisone is needed (11). After discontinuation of the treatment, around 70% of patients achieve normal UFC and about 10% may have sustained remission (11, 21).

A number of potential side effects have been associated with mitotane, including neurological side effects, anorexia, hypercholesterolemia, gastrointestinal symptoms, and abnormal liver function (38).

##### Etomidate

Etomidate blocks multiple steroidogenic enzymes including side-chain cleavage complex, aldosterone synthase, 17-hydroxylase, 11 beta-hydroxylase (CYP11B1), and 17–20 lyase enzymes (38).

Intravenous etomidate is often used in patients with severe hypercortisolism who cannot take oral medication (42). This drug is indicated in patients who are not immediate surgical candidates and for severe manifestations of CS such as hypertension, severe sepsis, or psychotic crises. Its use requires close monitoring at intensive care settings, for central nervous system depression. Close serum cortisol monitoring is indispensable to avoid AI. Etomidate use may be life-saving when all other treatments have failed (43).

##### Aminoglutethimide

Aminoglutethimide inhibits the conversion of cholesterol to pregnenolone (CYP11A1). Although cortisol decrease is gradual, some patients require replacement therapy with hydrocortisone. This drug has side effects that limit its use, including lethargy, sedation, dizziness, blurred vision, gastrointestinal discomfort, headache, myalgia, and skin rash (44). Aminoglutethimide is no longer used in patients with CD, due to the association with many unfavorable side effects (45).

##### Trilostane

Trilostane blocks steroidogenesis. This drug was effective for the treatment of CS in dogs. It did not show the same effectiveness in humans with CD (46). Trilostane is associated with major adverse effects including diarrhea, nausea, headache, asthenia, abdominal pain, paresthesias, and escape phenomenon. This drug is not used anymore for the treatment of CD, because of its limited efficacy, compared with other drugs (39).

#### GR Blockade

Glucocorticoid receptor blockade can be used in selected patients, but these drugs are associated with pharmacological interactions and adverse events related to their mechanism of action such as arthralgia, nausea, headache, peripheral edema, decreased blood potassium, endometrial thickening, and therefore require frequent monitoring. Moreover, they lack a reliable biomarker to monitor their efficacy; therefore, its use is limited.

##### Mifepristone

Mifepristone is a high-affinity non-selective GR antagonist that has greater affinity for the GR: 10 times compared with cortisol and 4 times compared with dexamethasone. It does not bind to the mineralocorticoid receptor (47). Its antagonism results in rapidly controlled systemic effects of cortisol excess in CS patients (48). Because the negative feedback is lost, leading to an increase in ACTH and subsequent cortisol levels, these parameters are not useful for monitoring the efficacy of the treatment or disease activity (47).

In a multicenter, open-label trial, mifepristone (at dose 300–1,200 mg daily) was given orally to 50 patients with CD associated with type 2 diabetes or hypertension, during a 6-month period. This drug showed an improvement of glycemic parameters (60% of patients) and diastolic blood pressure reduction (38% of patients) (ClinicalTrials.gov NCT00569582). The most common side effects are arthralgia, nausea, headache, peripheral edema, decreased blood potassium, fatigue, and endometrial thickening (22). The FDA approved mifepristone in 2012 and the specific indication is hyperglycemia or type 2 diabetes mellitus due to CS (22).

#### New Molecular Targets for CD in Clinical Trials

##### Osilodrostat (LCI 699)

Osilodrostat is an oral inhibitor of CYP11B1 that inhibits deoxycortisol hydroxylation to produce cortisol, the final step of its synthesis. The mechanism of action of this drug is similar to metyrapone (28).

In a 10-week, proof-of-concept study, this drug showed efficacy to normalize UFC in 11 of 12 patients with CD. It produces a rise in ACTH, 11-deoxycortisol, and 11-deoxycorticosterone that declined after discontinuation. Osilodrostat was generally well tolerated. Fatigue, nausea, and headache were the most frequent adverse events reported (23).

In a 22-week phase II trial in CD patients, 84 and 79% reached UFC normalization at 10 and 22 weeks, respectively (49). Plasma aldosterone and cortisol levels decreased in both studies, while an increase in the levels of their precursors was observed (23, 49).

Osilodrostat use as alternative treatment for CD is promising and two phase III studies are currently ongoing (50).

##### Levoketoconazole (COR-003)

Levoketoconazole is a drug in investigation for CS that acts similar to its enantiomer, KCN, but it is hypothesized to provide better efficacy and safety (50).

*In vitro* and *in vivo* studies showed that levoketoconazole might cause diarrhea, fatigue, dizziness, and abdominal pain. It might not be as damaging to the liver as KCN. Levoketoconazole could be used to treat endogenous CS of any cause in adult patients who are unwilling to have surgery, before surgery, or in whom surgery has failed, or while waiting for the effects of radiotherapy to occur. A phase III single-arm, open-label trial is currently ongoing to determine efficacy, safety, tolerability, and pharmacokinetics in CS patients (51). A phase III clinical trial to assess the safety and efficacy of levoketoconazole in the treatment of endogenous CS is being carried out (ClinicalTrials.gov Identifier: NCT03277690).

##### R-Roscovitine

R-roscovitine is a cyclin-dependent kinase (CDK) and cyclin E inhibitor that may be a potential therapeutic option for CD patients. R-roscovitine was first tested in a mouse model showing reduction of serum corticosterone and ACTH levels. R-roscovitine suppressed *in vitro* and *in vivo* ACTH expression, induced senescence and cell cycle exit in corticotroph tumor cells by overexpression of p27, p21, and p57, and downregulated cyclin E expression (52, 53). A phase II clinical trial in CD patients is ongoing.

#### Other Medical Prospective Treatments

##### Epidermal Growth Factor Receptor (EGFR)

Epidermal growth factor receptor family is considered a putative therapeutic target for CD. In murine studies, gefitinib (an inhibitor of EGFR tyrosine kinase activity) reduced tumor size and corticosterone levels, and reversed hypercortisolemia signs. EGFR signaling inhibition may be a novel strategy to treat CD, but further studies are needed (54, 55). A clinical trial is ongoing (ClinicalTrials.gov Identifier: NCT02484755).

##### Selective, Peptide Melanocortin 2 Receptor (MC2R) Antagonists

Adrenocorticotropic hormone acts on MC2R at the adrenal cortex and produces chronically increased circulating cortisol. A decrease in cortisol levels and adrenocortical androgen secretion (preserving mineralocorticoid hormone secretion) may be expected when the MC2R activation by ACTH is blocked. MC2R could therefore be a new target for the treatment of CD (56).

##### Chimeric Compounds

Chimeric compounds that bind to both D2R and SSTRs may act in synergy, with greater potency than each individual compound, to control ACTH release and tumor growth. SST/DA chimeras have the ability to interact with both receptors and present enhanced potency and efficacy than individual SSTR or DR agonists. Currently, second-generation chimeric compounds are under development (57). A recent study showed that the chimeric compound sst2/sst5/D2 (BIM-23A760) that acts through different molecular mechanisms, such as reduction of calcium concentration and inhibition of hormonal secretion on pituitary adenoma cells, represents a promising therapeutic tool (58).

##### Temozolomide Monotherapy and in Combination

Temozolomide is an oral alkylating chemotherapy agent, which is usually used for the treatment of high-grade astrocytoma, glioblastoma, and melanoma. This drug has shown promise as monotherapy and in combination with pasireotide, as a treatment for aggressive pituitary adenomas and carcinomas (7, 59).

Temozolomide causes deoxyribonucleic acid (DNA) damage because it depletes the DNA repair enzyme, O6-methylguanine-DNA-methyl transferase (MGMT), which results in tumor cell apoptosis. MGMT expression levels emerge as inversely related to therapeutic response to temozolomide. It has been used as a predictor of temozolomide effectiveness (60, 61). For aggressive corticotroph adenomas refractory to surgery, radiotherapy, or other medical treatment, temozolomide may represent an option (7).

##### Doxazosin

The selective alpha (1)-blocker doxazosin has shown inhibition of pituitary tumor cell proliferation in murine and human models and a decrease in plasma ACTH levels. There is not enough experience with this drug and further studies are needed (62).

##### MicroRNAs

MicroRNAs are small non-coding RNA molecules that regulate gene expression at the post-transcriptional level. MicroRNAs control different processes such us cell differentiation, cell growth, and apoptosis. Tumoral corticotroph cell proliferation may be limited through microRNAs genetic manipulation. It could therefore be a new treatment for CD, but future studies are necessary (63).

##### Monoclonal Antibodies

Bevacizumab, a monoclonal antibody, blocks the expression of vascular endothelial growth factor receptor 2. This receptor is overexpressed in aggressive pituitary tumors. Bevacizumab could be a novel target in the treatment of CD, decreasing cell proliferation in aggressive and fast-growing corticotroph tumors (64). Antiangiogenic therapy may be promising for the treatment of aggressive corticotroph tumors.

##### Rapamicin

The mTOR complex, a serine–threonine kinase, plays a key role in the cell growth, regulation, and proliferation. Rapamicin inhibits mTOR pathway and is a potential drug for the treatment of CD (65).

##### Testicular Orphan Nuclear Receptor 4 (TR4)

Overexpression of TR4 promotes corticotroph cell proliferation, ACTH secretion, and pro-opiomelanocortin (POMC) transcription. It could be another potential target to achieve control of hypercortisolism in patients with CD (66).

### Retinoic Acid

Retinoic acid is a product of the metabolism of vitamin A/retinol, whose most used derivative is its cis isomer called Isotretinoin.

For many years, retinoids have been applied to therapeutically treat skin diseases, mainly acne. As a consequence of increasing understanding of their molecular action, their uses were diversified, involving their application in oncology and non-cancer-related applications (premalignant conditions and diseases such as chronic obstructive pulmonary, Alzheimer, and Parkinson) (67). Some groups have reported antitumorigenic effects for retinoids in several malignant diseases: epithelial tumors (68), cutaneous malignant diseases (69, 70), Kaposi’s sarcoma (71, 72), squamous cell carcinomas (73, 74), cutaneous T-cell lymphoma (75, 76), neuroblastoma (77), lung cancer (78), acute promyelocytic leukemia (79, 80), and others. In addition, immune stimulating effects of RA have been described (81).

There is evidence that retinoids participate in pituitary development (82) and adult pituitary gland has retinoid receptors, specific genes with retinoic acid response element (RARE) sequence and enzymes of the metabolism of retinoids (83–86).

#### Mechanism of Action of RA

The classical mechanism of RA action involves a nuclear hormone receptor-mediated activation of the transcriptional machinery on target genes, as well as activation of kinase cascades. This ability to activate multiple transcriptional and non-genomic programs gives RA enormous therapeutic potential.

**Figure 1:**
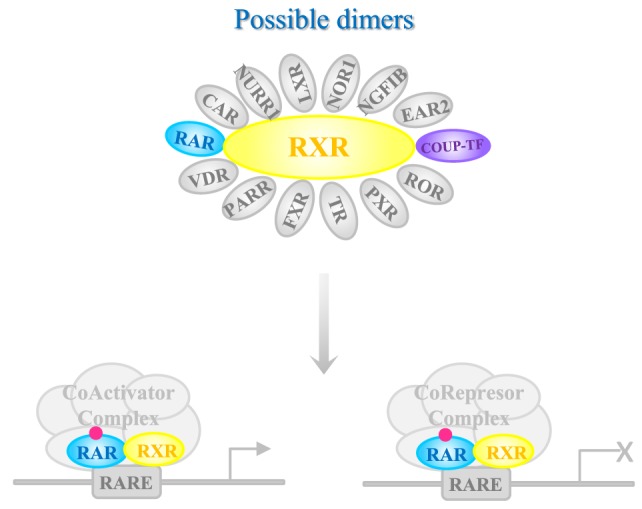
Mechanism of action of RA receptors (RARs). Retinoic acid (RA) nuclear receptor RARs and retinoid X receptors (RXRs) heterodimerize in the presence of the ligand that binds to RAR. The complex interacts with deoxyribonucleic acid containing specific RA responding element sequences [retinoic acid response element (RARE)], to mediate gene transcription. Chicken ovoalbumin upstream promoter transcription factor (COUP-TF) [as others nuclear receptors such as known ligand receptors vitamin D receptor (VDR), peroxisome proliferator-activated receptor (PPAR), liver X receptor (LXR), thyroid receptor, pregnane X receptor (PXR), RAR-related orphan receptor (ROR), and farnesol X receptor (FXR), or orphan receptors BGFIB, Nur77-related receptor 1 (NURR1), nuclear orphan receptor 1 (NOR-1), V-erbA-related protein 2 (EAR-2), and CAR] is able to dimerize with RXR. The complex with COUP-TF inhibits the effect of RA. Depending on the co-factors that are recognized by RAR-RXR complex, transcription of the target gene is activated or inhibited.

Once in the target tissue, RA is delivered to its nuclear receptor through intracellular binding proteins such as cellular retinol-binding protein type II and fatty acid-binding protein 5 (87). RA interacts with two classes of receptors, RA receptor (RAR) (88) or retinoid X receptor (RXR) (89), each of which has three isoforms (α, β, and γ) (Figure 1). Both receptors belong to the steroid hormone/nuclear receptor superfamily along with estrogen, thyroid hormone, vitamin D, and orphan receptors (90). RARs and RXRs heterodimers act as RA ligands, but RXRs can heterodimerize with other nuclear receptors [vitamin D receptor, PPAR, liver X receptor, thyroid receptor, pregnane X receptor, RAR-related orphan receptor, farnesol X receptor (FXR), chicken ovoalbumin upstream promoter transcription factor (COUP-TF), nerve growth factor inducible B, Nur77-related receptor 1 (NURR1), nuclear orphan receptor 1, V-erbA-related protein 2, and constitutively active receptor (CAR)] (91) that act as rexinoid ligands. These nuclear receptors can activate transcription through the RA response elements RAREs in the target genes or assemble into co-repressor complexes for other gene pathways (92). The large number of possible complexes brings a very high degree of plasticity (93, 94). Moreover, RAR/RXRs contain several phosphorylation sites modified by kinases such as protein kinase A, mitogen- and stress-activated protein kinase 1, CDKs, and mitogen-activated protein kinases, which further makes the response more complex. An additional level of complexity is the fact that the magnitude and duration of the response is regulated through RARs and RXRs degradation by the proteasome.

Chicken ovalbumin upstream promoter transcription factors belong to the superfamily of the steroid/thyroid hormone/vitamin receptors (95). COUP-TFs are supposed to have an important evolutionary and functional role given the high conservation of their amino acid sequence between species (96). COUP-TFs are orphan receptors because no endogenous ligands have been identified so far. COUP-TFI is more highly expressed in neuronal tissues of the central and peripheral nervous systems, whereas COUP-TFII is mainly present in developing organs (97–100).

Chicken ovoalbumin upstream promoter transcription factors are negative regulators of RA signaling (101–103). It has been reported that during development the signaling of COUP-TFs and retinoids have a functional crosstalk evidenced by the antagonistic action of COUP-TF on retinoid target genes and the fact that RA can modulate COUP-TFs expression (104, 105). In fact, COUP factors are considered as part of the RA signaling pathway (104–108). Besides, COUP-TFI, RARα, and RARβ knockout mice share common phenotypes (109–111).

#### RA in Corticotroph and Other Cellular Models

In AtT-20 pituitary ACTH-secreting tumor cells, an experimental cellular model of corticotroph adenomas, RA inhibits POMC transcription and stimulation of POMC by corticotropin-releasing hormone (CRH), being these effects highly specific because RA does not affect key factors of the corticotroph function such as cAMP response element-binding, GR, and paired-like homeodomain X 1 (112). In contrast to the action of RA showing POMC transcriptional inhibition, a synthetic agonist that acts only on RARα, Am80, has been described to increase POMC mRNA expression, CRH-induced ACTH secretion, and POMC promoter activity in AtT-20 corticotroph cells (113).

As part of its antitumorigenic action, RA inhibits ACTH and corticosterone-secreting cells proliferation. These antiproliferative effects involve transcription factors such as activator protein 1 and Nur77/Nurr1 (112). RA induces bone morphogenetic protein 4 (BMP-4), which participates in its antiproliferative effects (114) and has been also shown to be involved in the action of somatostatin analogs on corticotrophs (115). In GH3 and AtT-20 cells, RA-induced expression of BMP-4 involves chromatin remodeling (116). On the other hand, RA induces apoptosis/cell death in ACTH-secreting cells by inducing caspase-3 activity (112).

**Figure 2:**
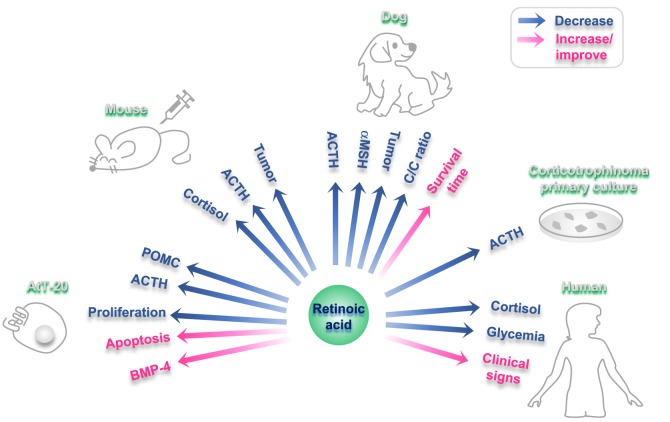
Effects of retinoic acid (RA) on different experimental models of Cushing disease. Blue arrows: hormones/factors/read-outs than decrease with RA administration; fuchsia arrows: hormones/factors/read-outs than increase/improve with RA administration.

Furthermore, RA inhibits ACTH production in primary culture of human corticotrophins (112). In accordance, RA reduces ACTH endogenous production in tumor cells although it has no effect on non-tumoral pituitary cells (112) (Figure 2). In primary cultures from 10 human adrenal specimens, RA blunted ACTH receptor transcription (117).

#### RA in Rodent and Canine Models

In RA-treated AtT-20 xenographed nude mice, an *in vivo* model of corticotropina, RA reduces tumor mass and reverses endocrine alterations (112). RA also decreases plasma ACTH and cortisol, together with a reduction in adrenal hyperplasia and skin atrophy in these rodents (112).

Cushing’s disease is a common disorder in dogs (118). A dose of 2 mg/kg body weight/day of RA results effective and safe in this model (119). This treatment with RA reduces plasma ACTH and melanocyte-stimulating hormone alpha and diminishes the cortisol/creatinine urine ratio, leading to normalization of the gonadal axis and the estrous cycle (119). After a period of 180 days of treatment, RA reduced pituitary adenoma size, while survival time and clinical signs (food intake, skin appearance, and hair recovery) improved (119). RA action has not adverse events neither hepatotoxicity in dogs (119) (Figure 2).

Besides, in a poodle dog with Nelson’s syndrome with iatrogenic hypoadrenocorticism induced by mitotane, RA reduced plasma ACTH and macroadenoma size after 10 months of treatment (2 mg/kg/day) (120).

#### RA in Humans

In the first study in seven patients with CD, in five patients UFC levels decreased markedly after RA administration for 6–12 months (doses of 10–80 mg/day) and three of them reached normalization (24). The levels of plasma ACTH did not show a modification at the end of the treatment in responsive patients, while other signs showed an improvement (blood pressure, glycemia, and sings of hypercortisolism). Adverse events were mild and transient, including conjunctive irritation, headache, arthralgia, and nausea. Three patients complained of mild arthralgias, another three of xerophthalmia, two of abdominal pain, and one patient reported headaches. There were no reports of hepatic or renal dysfunction. One of the responding patient maintained normal UFC levels after isotretinoin withdrawal. This would be explained by a prolonged inhibitory effect of RA on corticotroph tumor cells (24).

In a recently prospective trial, isotretinoin was given orally for 6–12 months to 16 patients with persistent or recurrent CD in a dose ranging from 20 to 80 mg/day. Before drug administration and every month, clinical/biochemical data were registered. An initial response with normalization of the UFC and salivary cortisol was observed in six patients (37.5%). Subsequently, two patients escaped or relapsed. The group of responding patients had significantly lower mean age and lower mean basal levels of UFC, nocturnal salivary cortisol, and ACTH, in comparison with non-responders patients. Only patients who had UFC levels <2.5-fold ULN obtained UFC normalization. In a non-responding patient treated with a full-tolerated dose of CAB (3 mg/week), UFC levels were normalized after the addition of isotretinoin 50 mg/day. This combination allowed the reduction of CAB dose to 2 mg/day and hence the optimization of the tolerance profile. At the end of the study, four patients (25%) achieved the normalization of UFC levels. In the rest of the patients, UFC levels experienced up to 52.1% reduction. Isotretinoin side effects were experienced by seven patients (conjunctiva irritation, cheilitis, mucositis, nausea, headache, and arthralgias), though they were mostly transient. The authors of this study conclude that RA could be useful in patients with mild hypercortisolism (25).

The FDA approved RA for acne treatment in 1982. The usual dose for the treatment of acne vulgaris is 0.5–1 mg/kg/day. In patients with CD, in previous clinical trials, the initial dose was 20 mg/day and the maximum dose was 80 mg/day.

The described mild adverse effects for acne treatment include skin and annexes disorders, xerophthalmia, dry skin, conjunctiva irritation, dermatitis, pruritus, rash, hair fragility, and alopecia. Musculoskeletal system disorders: pain in muscle and joints, arthritis. Sensory disorders: photophobia, lenticular cataract, and keratitis. Gastrointestinal system disorders: nausea, intestinal inflammatory diseases such as colitis, ileitis, and hemorrhages. Laboratory findings: transaminases elevation (reversible and transient), increase in serum triglycerides and cholesterol, hyperuricemia. Although a causal relationship has not been established, high fasting blood sugar levels have been reported, and new cases of diabetes have been diagnosed during therapy. Serious adverse effects included psychiatric disorders, rash, metabolic disorders, increased intracranial pressure, renal, and abdominal disturbances. RA is contraindicated in patients with renal or hepatic insufficiency.

Retinoic acid is highly teratogenic; therefore, it is contraindicated not only in pregnant women but also in women of childbearing age. The latter group could only use RA in combination with two contraception methods (121).

In the two studies on patients with CD, RA has shown good response pattern in patients with mild hypercortisolism. It was generally well tolerated with transient and mild adverse effects (arthritis, xerophthalmia, nausea, and headache) (24, 25). This would represent an advantage, over other drugs such as pasireotide (worsening diabetes symptoms), KCN (hepatotoxicity), and mifepristone (arthralgia, peripheral edema, decreased blood potassium, fatigue, and endometrial thickening).

## Future Perspectives

In primary culture of human corticotrophins, normal ACTH-secreting cells express COUP-TFI, but tumoral cells do not (112). Corticotroph normal cells do not respond to RA treatment and this may be attributed to the fact that they express the inhibitory factor COUP-TFI. In fact, when tumoral cells responding to RA are transfected with a COUP-TFI expression vector, the response to RA is abolished (112). In 34 specimens of human pituitary adenomas, 29 expressed COUP-TFI but only 5 of them presented expression of COUP (122). This interesting difference between normal/tumoral COUP-TFI expression makes this protein a promising biomarker of a possible response of the cells to RA action. In lung cancer, the antiproliferative action of RA depends on Nur77/COUP-TFI balance (123). Currently, there are no long-term studies involving RA. An ongoing controlled clinical trial in which both, the long-term efficacy of RA and the correlation with the expression of COUP-TFI are tested, would contribute to clarify the future perspective to implement RA as a potential drug for CD treatment.

## Conclusion

Cushing’s disease presents high morbidity and mortality in the absence of proper treatment.

Although transsphenoidal surgery is the primary option, some patients are not candidates to surgery, or do not achieve remission after long-term control.

New knowledge about the mechanisms involved in the pathophysiology of CD led to the discovery of new targets for developing drugs that seek to reduce the secretion of ACTH. Targeting specific molecules, and searching biomarkers which predict response to different drugs, is the path to accomplish effective treatment. RA represents a new alternative to medical treatment in CD, but the true efficacy should be assessed in long-term controlled studies.

## Author Contributions

MF, JR, MG, and EA conducted literature review and wrote the manuscript. EA and MG conceived the concept and idea of this article. JT and LN contributed to review, discussion, and conception of the work. MG and EA have critically read, revised, and edited the manuscript.

## Conflict of Interest Statement

The authors declare that the research was conducted in the absence of any commercial or financial relationships that could be construed as a potential conflict of interest.

## Abbreviations

α-MSH, melanocyte-stimulating hormone alpha; ACTH, adrenocorticotropic hormone; AI, adrenal insufficiency; AP-1, activator protein 1; BMP-4, bone morphogenetic protein 4; CAB, carbergoline; CAR, constitutively active receptor; CD, Cushing’s disease; CDK, cyclin-dependent kinase; COR-003, levoketoconazole; COUP-TF, chicken ovoalbumin upstream promoter transcription factor; CRBPII, cellular retinol-binding protein type II; CREB, cAMP response element-binding; CRH, corticotropin-releasing hormone; CS, Cushing’s syndrome; CYP, cytochrome P450; CYP11B1, 11 beta-hydroxylase; D2, dopamine receptor 2; DNA, deoxyribonucleic acid; DR, dopamine receptor; EAR-2, V-erbA-related protein 2; FABP5, fatty acid-binding protein 5; FDA, Food and Drug Administration; FXR, farnesol X receptor; GR, glucocorticoid receptor; KCN, ketoconazole; LCI699, osilodrostat; LXR, liver X receptor; MAPK, mitogen-activated protein kinase; MC2R, melanocortin 2 receptor; MGMT, O6-methylguanine-DNA-methyl transferase; MSK1, mitogen- and stress-activated protein kinase 1; NGFIB, nerve growth factor inducible B; NOR1, nuclear orphan receptor 1; Nur77, nerve growth factor IB; NURR1, Nur77-related receptor 1; OPDD, mitotane; PKA, protein kinase A; POMC, pro-opiomelanocortin; PPAR, peroxisome proliferator-activated receptor; PTX1, paired-like homeodomain X 1; PXR, pregnane X receptor; RA, retinoic acid; RAR, retinoic acid receptor; RARE, retinoic acid response element; ROR, RAR-related orphan receptor; RXR, retinoid X receptor; SOM230, pasireotide; SRL, somatostatin receptor ligand; SSTR, somatostatin transmembrane receptor; TR, thyroid receptor; UFC, urinary free cortisol; VDR, vitamin D receptor.
